# Haemoglobin D-Punjab haemoglobinopathy, the silent epidemic: A prospective hospital based study in Gujarat, India

**DOI:** 10.6026/973206300220015

**Published:** 2026-01-31

**Authors:** Garima Anandani, Parth Goswami, Vaishali Bhankhodia, Sagar Dholariya, Yash Thesiya, Avani Dangar

**Affiliations:** 1 Department of Pathology, AIIMS, Rajkot, Gujarat, India; 2 Department of Biochemistry, AIIMS, Rajkot, Gujarat, India

**Keywords:** Gujarat, Haemoglobinopathy, HbD-Punjab, High-performance liquid chromatography

## Abstract

Haemoglobin D-Punjab (HbD-Punjab) is a globally prevalent variant that remains under-recognized in the Saurashtra-Kutch region of Gujarat. Therefore, it is of interest to determine its prevalence using High-Performance Liquid Chromatography (HPLC) and to evaluate associated clinical and haematological features. A cross-sectional analysis of 22 confirmed HbD cases revealed predominantly heterozygous HbD-Punjab, with a marked female predominance and mixed symptomatic presentation. HbD percentages showed no meaningful correlation with haemoglobin concentration, red cell indices, or iron parameters. Thus, we show the need for broader population-based screening to improve early detection and management of HbD haemoglobinopathy in Gujarat.

## Background:

There exists a substantial diversity in the prevalence of haemoglobinopathies around the world. In India, it is estimated that 0.37 per 1,000 fetuses are diagnosed with this disorder [1]. The significant occurrence of genetic abnormalities in India is a result of the high birth rate, the vast population, and the cultural preference for marrying within families among diverse communities [2, 3]. The most prevalent haemoglobinopathy documented in India is the β-thalassemia trait (BTT) [4, 5]. This is also true for Gujarat, where it stands out as the leading haemoglobinopathy [6, 7]. Research conducted in the Saurashtra-Kutch area of Gujarat indicated that 11.26% of the population had various haemoglobinopathies. Of these individuals, 40% were diagnosed with the BTT, 5.5% with the Haemoglobin (Hb) D-Punjab trait, 3.2% with the sickle cell trait, and 1.7% with the δβ-thalassemia trait or hereditary persistence of fetal haemoglobin (HPFH) trait. Other haemoglobinopathies were observed in lesser frequencies, accounting for 0.15% [8]. Hb D includes numerous variants, with Hb D-Punjab standing out as the most frequently encountered variant [9]. The Hb D-Punjab variant is the result of a point mutation occurring in the beta-globin gene (HBB) at the first base of the 121 codon (GAA → CAA). This alteration leads to the replacement of glutamic acid (Glu) with glutamine (Gln) within the HBB (HBB: c.364G>C) [10]. It is predominantly observed in individuals of Punjabi, Northern European, Chinese, Turkish, African-American, Greek and Yugoslav descent [11]. Compared to Punjabis, the Hb D Punjab trait is a less prevalent haemoglobinopathy among Gujaratis [12]. There are four distinct forms of Hb D: the heterozygous Hb D trait, HbS-D disease, Hb D-thalassemia, and homozygous Hb D disease [11]. In the majority of instances, variations of HbD present either as asymptomatic or in mild to moderately severe forms, characterized by symptoms such as lethargy, weakness, and symptomatic haemolytic anemia [13]. Therefore, it is of interest to investigate the distribution of HbD Haemoglobinopathy through HPLC technique conducted at a tertiary care centre in Rajkot, and to examine the spectrum of HPLC findings, red blood cell (RBC) indices and clinical features in HbD Haemoglobinopathy, determine their correlation and statistical significance as screening tests in identifying these cases.

## Materials and Methods:

This research is a cross-sectional analytical study based on clinical and laboratory evaluations carried out in the Department of Pathology at a tertiary care facility from June 2024 to January 2024. This study is a part of an institutional intramural funded project which was approved by institutional ethics committee. Written informed consent is taken from all the participants, and from their guardians if age is less than 18 years. The study encompassed samples submitted for Hb HPLC testing that were subsequently identified as having HbD haemoglobinopathy. The participants included individuals suspected of having thalassaemia syndromes or haemoglobinopathies, regardless of age; antenatal cases along with their relatives seeking prenatal testing for these conditions; individuals voluntarily opting for pre-marital testing for thalassaemia syndromes or haemoglobinopathies; and patients with a confirmed family history of thalassaemia syndromes or haemoglobinopathies. The exclusion criteria were restricted to individuals who had already received a diagnosis of thalassaemia syndromes or haemoglobinopathies. Amongst these, cases confirmed to have HbD haemoglobinopathy through HPLC were included in the study, comprising of 22 cases. The clinical manifestations of the patients were recorded; complete blood count (CBC) was performed using the Sysmex XN 1000, an electronic automated six-part haematology analyser. Follow by HPLC with the ARKRAY ADAMS HA-8180T automated HPLC analyser.

## Statistical analysis:

Data were analysed using descriptive statistics i.e., mean ± standard deviation (SD) or median / inter-quartile range (IQR) and inferential statistics i.e., Pearson's correlation for parametric data and Spearman's rank correlation for non-parametric data. Statistical significance was set at p < 0.05. All analyses were performed using Statistical Package for Social Sciences (SPSS) version 28.0 and Microsoft Excel.

## Results:

**Figure 1:**
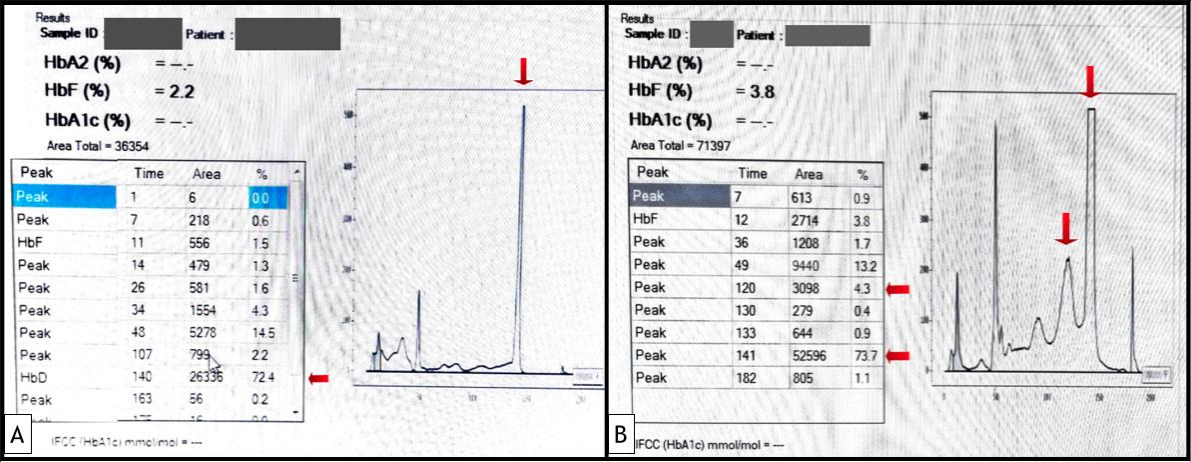
(A) HPLC chromatogram showing an abnormal peak of 72.4% at a retention time (RT) of 140 seconds that is D window, HbF and HbA2 of 2.2% each, suggestive of homozygous HbD-Punjab. (B) HPLC chromatogram showing an abnormal peak of 73.7% in the D window, HbA2- 4.3% and HbF 3.8% suggestive of HbD Punjab-β thalassemia.

Total 2673 cases were run on HPLC of which 2186 were normal and 487 were abnormal showing thalassemia syndrome or haemoglobinopathies. Amongst these positive cases, 22 (4.51%) were diagnosed to have HbD haemoglobinopathy. Of these, 20 were HbD Punjab heterozygous, one was homozygous HbD Punjab, and one double heterozygous for HbD Punjab with β thalassemia based on HPLC findings (Figure 1). The study analysed the distribution of clinical features and symptom prevalence among different HbD Punjab variants (Table 1).

The findings revealed that females were predominantly affected, constituting 86% of the total cases, while males accounted for only 14%. Within the heterozygous HbD Punjab group, 95% of the cases were females, highlighting a significant gender disparity. Among the total cases, 59% were symptomatic, while 41% were asymptomatic. All asymptomatic cases belonged to the heterozygous HbD Punjab group, suggesting that this variant may often present without noticeable symptoms. The most common symptoms reported were fatigue and weakness, affecting 50% and 55% of the total cases, respectively. These symptoms were predominantly observed in the heterozygous HbD Punjab group. In contrast, less common symptoms such as pedal edema, mild hepatosplenomegaly, and mild haemolytic anemia were observed in only 9-14% of the total cases. Notably, these rarer symptoms were primarily seen in the heterozygous HbD Punjab and HbD Punjab with β thalassemia groups. Hence, the study highlights that fatigue and weakness are the most prevalent symptoms in heterozygous HbD Punjab patients, particularly in the heterozygous variant. While severe symptoms are rare, they may be more common in patients with HbD Punjab with β thalassemia. The findings underscore the need for larger studies to better understand the clinical spectrum of these variants and to improve diagnostic and management strategies. The descriptive analysis of haematological parameters was done according to the normal reference ranges, in 20 cases of heterozygous HbD which revealed several key findings (Table 2) [14].

Mean ± standard deviation (SD) was calculated for the parametric data, while median / interquartile range (IQR) was calculated for the non-parametric data. RBC- red blood cell, PCV- packed cell volume, Hb- haemoglobin, MCV- mean corpuscular volume, MCH- mean corpuscular haemoglobin, MCHC- mean corpuscular haemoglobin concentration, RDW-CV- red cell distribution width- coefficient of variance, WBC- white blood cell, PLT- platelet, PDW-platelet distribution width, MPV- mean platelet volume, P-LCR- platelet large cell ratio, PCT- plateletcrit. The median age of the patients was 27 years, indicating a relatively young population. The median Hb level was 10.7 g/dl, which is significantly lower than the normal reference range, confirming the presence of mild to moderate anemia. The mean corpuscular volume (MCV) was 75.6 ± 10.2 fL, below the normal range of 80-100 fL, suggesting microcytic anemia. This is further supported by the mean corpuscular haemoglobin (MCH) value of 24.6 ± 4.3 pg, which is also below the normal range of 27-34 pg. The red cell distribution width- coefficient of variance (RDW-CV) had a median of 15.4%, which is elevated compared to the normal range of 11.5-14.5%, indicating significant anisocytosis. This is consistent with the presence of a haemoglobinopathy. The Mentzer's index had a median of 18.3, which is above the cutoff of 13, ruling out thalassemia as the primary cause of microcytosis. The HbD percentage was 37.5 ± 3.8%, which is expected in heterozygous HbD cases, as normal individuals do not have HbD. The HbA2 level was 1.95 ± 0.25%, within the normal range of 2-3.5%, ruling out concomitant β-thalassemia. The median HbF level was 0.3%, which is within the normal range for adults (<1%). The serum ferritin levels had a median of 18.9 ng/ml, which falls within the normal reference range for individuals <50 years (6.24-137.0 ng/ml). This suggests that iron deficiency is unlikely to be a contributing factor to anemia in this cohort. The correlation analysis revealed that HbD percentage does not show any significant relationship with other laboratory parameters, as all p-values were greater than 0.05 (Table 3).

RBC- red blood cell, PCV- packed cell volume, Hb- haemoglobin, MCV- mean corpuscular volume, MCH- mean corpuscular haemoglobin, MCHC- mean corpuscular haemoglobin concentration, RDW-CV- red cell distribution width- coefficient of variance, WBC- white blood cell, PLT- platelet, PDW-platelet distribution width, MPV- mean platelet volume, P-LCR- platelet large cell ratio, PCT- plateletcrit. This indicates that HbD percentage operates independently of haematological and biochemical indices in individuals with heterozygous HbD. In other words, the level of HbD in the blood does not directly influence or correlate with Hb levels, RBC indices, or iron status. Hence, the lack of significant correlations between HbD percentage and other haematological parameters in heterozygous HbD cases can be attributed to the mild nature of the condition. Heterozygous HbD typically presents with minimal haematological abnormalities, as the presence of normal HbA compensates for the abnormal HbD, resulting in near-normal RBC indices and Hb levels. This explains why HbD percentage does not strongly influence other laboratory parameters, highlighting its independent role in the pathophysiology of the condition.

## Discussion:

Haemoglobinopathies refer to genetic disorders that cause the development of abnormal variants of Hb. The majority of these conditions stem from mutations affecting the alpha or beta globin chains, leading to changes in the structure of Hb molecules. Generally, they do not present with clinical symptoms in heterozygous individuals; however, they may result in severe anemia when in homozygous states or in conjunction with thalassemias [15]. Hb D-Punjab ranks among the most frequently occurring Hb variants globally, following Hb S and Hb C. It is particularly common in the Punjab region of Northwest India, where its estimated prevalence is approximately 2.0% which is reduced to 1% in western India [16]. HbD-Punjab disease can develop in individuals with a single copy of the HbD mutation or in those who possess a combination of the HbD mutation and other genetic modifications affecting their Hb. Studies have shown that the prevalence of heterozygosity for HbD-Punjab is around 1.1% in North India [17]. Some patients with HbD, both homozygous and heterozygous forms, may remain asymptomatic and demonstrate a normocytic normochromic appearance in peripheral blood smear evaluations. However, others could experience moderate symptoms along with microcytic hypochromic anemia [18, 19]. The presence of low RBC indices in HbD haemoglobinopathy, as detected by HPLC, could be attributed to the co-inheritance of alpha deletion or beta mutation, or it may be associated with iron deficiency anemia. HbS-D disease, characterized by the combination of Hb D trait and sickle cell disease, manifests clinical symptoms that are similar to those seen in sickle cell disease [20, 21]. Hb D-thalassemia refers to the inheritance of the Hb D trait in conjunction with β- thalassemia. Clinically, individuals affected by Hb D-thalassemia may experience mild microcytic anemia, with the severity dependent on the degree of β-thalassemia mutations, as well as various complications linked to thalassemia. Additionally, Hb D-thalassemia leads to anemia resulting from diminished production of HbA, which can cause symptoms such as fatigue and weakness. The condition may also result in splenomegaly due to the premature destruction of fragile abnormal RBCs, thereby increasing spleen activity. Jaundice may develop as a consequence of RBC breakdown, and persistent fatigue is often observed due to the reduced capacity for oxygen transport [20, 22]. Homozygous Hb D disease, known as Hb DD disease, is marked by mild haemolytic anemia and persistent, non-progressive splenomegaly. Notably, it typically does not require any specific treatment and is associated with minimal health complications [20]. Unlike alkaline and acid electrophoresis, on HPLC, it elutes in the specific D-Window. Hence this is a sensitive method to identify this Hb variant [23, 24]. The differential diagnosis for homozygous HbD disease includes HbD-β thalassaemia. It is essential to distinguish between these two conditions by evaluating the RBC indices, as well as the levels of HbA2 and HbF. A significant concern in excluding HbD-β thalassaemia is that while homozygous HbD disease typically results in mild haemolytic anaemia, the co-inheritance of β thalassaemia can lead to a more severe form of chronic haemolytic anaemia [25]. On HPLC, HbD-β+ thalassaemia often indicate an elevated HbD, alongside an HbA2 level greater than 3.9%. In cases of double heterozygosity for HbD Punjab and β0 thalassaemia, there is a major HbD band representing 80-90% of the total Hb, with a mild increase in HbF levels between 3-6%, and the absence of an HbA0 peak [8]. In a study conducted by Pant *et al.* involving 4,800 cases, haemoglobinopathies were identified in 290 individuals, representing 6.04% of the total cases, as determined by CE-HPLC. Among these, 15 cases were identified as heterozygous HbD Punjab, with Hb D levels between 31% and 40% alongside nearly normal RBC parameters. Additionally, four cases were found to be double heterozygous Hb D-β thalassaemia trait, with HbD levels ranging from 77% to 81.3% and elevated HbA2 levels between 3.8% and 6%. Notably, no instances of homozygous HbD Punjab were reported [26]. Sachdev *et al.* conducted a study involving 2,600 cases, identifying haemoglobinopathies in 327 instances. Within this group, 0.5% of the cases were HbD-Punjab heterozygous, diagnosed through CE-HPLC. Notably, the researchers did not encounter any case of HbD-Punjab homozygous [27]. Srinivas *et al.* identified haemoglobinopathies in 0.55% of the cases examined in their study with 7.8% showing HbD Punjab haemoglobinopathy, comprising of 23 cases of heterozygous, nine homozygous, two as HbS/D, and four as HbD/β thalassaemia [26]. For asymptomatic individuals possessing the HbD trait and HbDD, it is essential to engage in regular consultations with a healthcare professional to monitor Hb levels. Genetic counseling is advisable to gain insights into inheritance patterns and to assist in family planning. Prenatal screening should be contemplated for those intending to conceive. Generally, individuals with the HbD trait can maintain a healthy lifestyle with proper monitoring and awareness. In HbSD disease scenarios, consistent medical follow-ups are vital, and treatment options may include hydroxyurea therapy, blood transfusions, and stem cell transplantation, contingent upon the severity of the condition [22]. The sequencing of DNA from alpha and beta globin genes is used for confirming Hb variants, particularly point mutations [15]. In premarital and prenatal screening, molecular testing is frequently not accessible, hence, Hb HPLC analysis, family studies, and epidemiological data being the reliable diagnostic methods [9].

## Conclusion:

HbD-Punjab haemoglobinopathy, though generally mild, carries significant reproductive implications when co-inherited with variants such as HbS or β-thalassemia, necessitating targeted family studies and genetic counseling. The disorder's minimal haematological impact underscores the need for proactive screening rather than reliance on clinical presentation. Strengthening population-level awareness and implementing comprehensive premarital and antenatal screening programs are essential to prevent severe double-heterozygous states and guide future public health policy.

## Funding:

Part of an intramural funded project of AIIMS Rajkot

## Disclosure of ethical statements:

[1] Approval of the research protocol: Approved by institutional ethics committee of AIIMS Rajkot. Approval number is AIIMS. Rajkot/IEC/47/2023 dated 13-09-2023.

[2] Informed Consent: Written informed consent is taken from all the participants, and from their guardians if age is less than 18 years.

[3] CTRI Registration No. of the study/trial: CTRI/2024/05/067966

## Figures and Tables

**Table 1:** Distribution of clinical features and symptom prevalence in HbD Punjab variants

**Type of HbD Punjab Variant**	**Total Cases**	**Males**	**Females**	**Asymptomatic**	**Symptomatic**	**Fatigue**	**Weakness**	**Pedal Edema**	**Mild Hepatosplenomegaly**	**Mild Haemolytic Anemia**
Heterozygous HbD Punjab	20	1 (5%)	19 (95%)	9 (45%)	11 (55%)	9 (45%)	10 (50%)	2 (10%)	2 (10%)	2 (10%)
Homozygous HbD Punjab	1	1 (100%)	0 (0%)	0 (0%)	1 (100%)	1 (100%)	1 (100%)	0 (0%)	0 (0%)	0 (0%)
HbD Punjab with β Thalassemia	1	1 (100%)	0 (0%)	0 (0%)	1 (100%)	1 (100%)	1 (100%)	0 (0%)	1 (100%)	1 (100%)
Total	22	3 (14%)	19 (86%)	9 (41%)	13 (59%)	11 (50%)	12 (55%)	2 (9%)	3 (14%)	3 (14%)

**Table 2:** Descriptive statistics of haematological parameters in heterozygous HbD cases

**Parameter**	**Mean ± SD OR Median (IQR)**	**Normal Reference Interval**
Age (years)	27 (41) *	N/A
RBC count (million/cumm)	4.34 ± 0.72	Males: 4.7-6.1; Females: 4.2-5.4
Hb (g/dl)	10.7 ± 2.2	Males: 13.8-17.2; Females: 12.1-15.1
PCV (%)	32.9 ± 6.1	Males: 38.3-48.6; Females: 35.5-44.9
MCV (fL)	75.6 ± 10.2	80-100
MCH (pg)	24.6 ± 4.3	27-34
MCHC (g/dl)	31.9 ± 2.1	32-36
RDW-SD (fL)	40.6 (2.6) *	39-46
RDW-CV (%)	15.4 (3.1) *	11.5-14.5
Mentzer's index	18.3 (6.6) *	<13 suggests thalassemia
WBC Count (x10^3^/µL)	7.54 (2.11) *	11-Apr
PLT (x10^3^/µL)	281 (81) *	150,000-450,000
PDW (fL)	11.2 (5.5) *	9-14
MPV (fL)	10.3 (1.8) *	7.5-11.5
P-LCR (%)	25 (14.6) *	15-35
PCT (%)	0.29 (0.11) *	0.15-0.35
HbA0 (%)	52.1 ± 4.5	95-98
HbF (%)	0.3 (0.4) *	<1 (adults)
HbA2 (%)	1.95 ± 0.25	2-3.5
HbD (%)	37.5 ± 3.8	Absent
S. Ferritin (ng/ml)	18.9 (19.4) *	< 50 years; 6.24- 137.0 ng/ml
		≥ 50 years: 11.1-264.0 ng/ml
*is Median (IQR)

**Table 3:** Correlation of HbD (%) with other laboratory parameters

**Parameter**	**Correlation Coefficient (r)**	**p-value**
Age	-0.08†	0.73
RBC count (million/cumm)	-0.15*	0.53
Hb (g/dl)	0.10*	0.67
PCV (%)	0.09*	0.7
MCV (fL)	0.05*	0.83
MCH (pg)	0.08*	0.73
MCHC (g/dl)	0.14*	0.56
RDW-SD (fL)	-0.18†	0.45
RDW-CV (%)	-0.20†	0.4
Mentzer's index	-0.12†	0.62
WBC count (/cumm)	-0.10†	0.67
Plt (/cumm)	-0.14†	0.56
PDW (fL)	-0.16†	0.5
MPV (fL)	-0.11†	0.65
P-LCR (%)	-0.13†	0.59
PCT (%)	-0.15†	0.53
HbA0 (%)	-0.22*	0.35
HbF (%)	-0.10†	0.67
HbA2 (%)	0.18*	0.45
Serum Ferritin (ng/ml)	0.12†	0.62
*Pearson's correlation and †Spearman's rank correlation
